# Association between *Helicobacter pylori* infection and metabolic syndrome and its components

**DOI:** 10.3389/fendo.2023.1188487

**Published:** 2023-06-19

**Authors:** Ying Liu, Ping Shuai, Wanjing Chen, Yuping Liu, Dongyu Li

**Affiliations:** ^1^ Department of Health Management Center & Institute of Health Management, Sichuan Provincial People’s Hospital, University of Electronic Science and Technology of China, Chengdu, China; ^2^ Chinese Academy of Sciences Sichuan Translational Medicine Research Hospital, Chengdu, Sichuan, China

**Keywords:** metabolic syndrome, *Helicobacter pylori*, obesity, hypertension, hyperglycemia

## Abstract

**Background and aim:**

The association between *Helicobacter pylori* (*H. pylori*) infection and metabolic syndrome (MetS) has been studied previously; however, the results remain controversial, which could be partly due to the different criteria used for defining MetS. We adopted five MetS criteria to provide better understanding of the association between *H. pylori* infection and MetS.

**Methods:**

Physical examination data of 100,708 subjects were obtained from January 2014 to December 2018. MetS was defined based on five criteria including: International Diabetes Federation (IDF), The Third Report of the National Cholesterol Education Program Expert Panel, Adult Treatment Panel III (ATP III), Joint Statement of International Multi-Societies (JIS), Chinese Diabetes Society (CDS), and the Guidelines for the Prevention and Treatment of Type 2 Diabetes in China (2017 edition)(CDS DM). Multivariate logistic regression analysis was performed to elucidate the association between *H. pylori* infection and MetS and its components.

**Results:**

The prevalence of MetS defined assessed using IDF, ATP III, JIS, CDS and CDS DM criteria was 15.8%, 19.9%, 23.7%, 8.7% and 15.4%, respectively. In males, the prevalence of MetS assessed using the five criteria in *H. pylori*-positive group was higher than that in negative-group; however, in females, same results were obtained using the three international criteria. In males, the prevalence of all MetS components was found to be higher in the *H. pylori*-positive group than those in the negative group; however, in females, only the prevalence of dyslipidemia and waist circumferences exhibited significant differences. Multivariate logistic regression analysis revealed that *H. pylori* infection in males was positively correlated with MetS. Additionally, *H. pylori* infection was found to be positively correlated with the waist circumference in the general population, and with hypertension and hyperglycemia in males.

**Conclusions:**

*H. pylori* infection was found to be positively associated with MetS in males in China.

## Introduction

Metabolic syndrome (MetS) is a major health issue in the contemporary society. It is reported that about 20-25% of adults worldwide suffer from MetS, which is a syndrome associated with the accumulation of multiple metabolic risk factors in individuals (1). Although MetS is an outcome of the interaction between multiple genetic and environmental risk factors, insulin resistance (IR) is considered as the key factor associated with MetS (2). The disorders of glucose, lipid, and protein metabolism are all associated with IR and eventually lead to obesity, cardiovascular disease, non-alcoholic fatty liver, and other related metabolic diseases (3). Therefore, Stern (4) proposed the “common soil” theory to explain the basic role of IR and effects of IR on multiple organs and systems, and evealing the factors that promote IR will help to understand the mechanism of MetS formation. In recent years, the accumulating evidence indicates that gastrointestinal microbes play a significant role in IR development (5). Moreover, previous studies have revealed that gut microbes are associated with MetS components such as diabetes, hypertension, and obesity (6–8), which would link gastrointestinal microbes to the development of MetS.

*Helicobacter pylori* (*H. pylori*), as an important and widely concerned gastrointestinal microbe, has a high infection rate worldwide. The infection rate of *H. pylori* is considerably high in developing countries, and up to 50% in China (9). In addition to the well-known association with chronic gastritis, gastric ulcer, and gastric cancer, growing attention has been paid to the extra-gastrointestinal effects of *H. pylori* infection, which has been found to impaired insulin signaling in hepatocytes, and increase the expression of a variety of inflammatory factors involved in IR development that has been related to MetS (10, 11). Based on this potential pathogenesis link mentioned above, researchers are interested in exploring the correlation between *H. pylori* infection and MetS and its components.

Some studies have attempted to describe the possible correlation between *H. pylori* infection and MetS, but the results are inconsistent and conflicting (12–15). Polyzos et al. indicated that the inconsistency of the results may be partly attributed to the different criteria used for defining MetS and different methods used for the detection of *H. pylori* infection (16). As known, different criteria with different parameters were developed to identify MetS, such as International Diabetes Federation (IDF) (17), Third Report of the National Cholesterol Education Program Expert Panel, Adult Treatment Panel III (ATP III) (18), Joint Statement of International Multi-Societies (JIS) (19), Chinese Diabetes Society (CDS) (20), Guidelines for the Prevention and Treatment of Type 2 Diabetes in China (2017 edition) (CDS DM) (21). Meanwhile, the methods for detecting *H. pylori* infection vary among different studies, including the specific antibody of *H. pylori* in serum and ^13^C urea breath test (UBT). When discussing the association between *H. pylori* infection and MetS, it is worth investigating whether applying different criteria for MetS would affect the results. In our study, *H. pylori* infection was determined using the UBT, which is highly recommended to conduct non-invasive screening of *H. pylori*, and MetS was defined using different international and Chinese criteria mentioned-above that are widely accepted. Using this strategy, we aimed to improve our understanding on the association between *H. pylori* infection and MetS and its components after excluding the influence of differing criteria.

## Materials and methods

### Design of the study

This was a retrospective cross-sectional study conducted at the Health Management Center of Sichuan Provincial People’s Hospital (Chengdu, China) from January 2014 to December 2018. The subjects were categorized based on their sex. The differences in the prevalence of MetS and its components were compared between the *H. pylori*-positive and -negative groups. Multivariate logistic regression analysis was performed to elucidate the association between *H. pylori* infection and MetS and its components.

### Participants

All subjects were asked to complete a medical history questionnaire, followed by physical examination (height, body weight, blood pressure, circumference of waist, hip, and neck), and laboratory examination (blood routine test, liver and kidney function, fasting plasma glucose [FPG], hemoglobin A1c [HbA1c], uric acid [UA], total cholesterol [TC], triglycerides [TG], low-density lipoprotein cholesterol [LDL-C], high-density lipoprotein cholesterol [HDL-C]), abdominal color Doppler ultrasound, chest imaging (X-ray or CT), and ^13^C urea breath tests (UBT).

Subjects were excluded if they exhibited the following: (a) history of gastrectomy or subtotal gastrectomy; (b) unable to perform ^13^C urea breath test due to pregnancy, lactation or other reasons; (c) immune system diseases, severe heart, liver and kidney dysfunction, and tumor patients; (d) history of anti-*H. pylori* therapy in the past three months.

### Criteria of MetS

Five criteria were used to define MetS, including IDF, ATP III, JIS, CDS, and CDS DM. In IDF, ATP III and JIS criteria, the standards applicable to Chinese were adopted, as shown in **Table 1**.

**Table 1:** The definition of MetS.

Items		IDF	ATP III	JIS	CDS	CDS DM
A						
Waist circumference (cm)	male	≥ 90	≥ 90	≥ 85	N/A	≥ 90
	female	≥ 80	≥ 80	≥ 80	N/A	≥ 85
B anti-hypertensive treatment						
or blood pressure (mmHg)		≥ 130/85	≥ 130/85	≥ 130/85	≥ 140/90	≥ 130/85
C T2DM previously diagnosed or use of any hypoglycemic drugs						
or FPG (mmol/L)		≥ 5.6	≥ 5.6	≥ 5.6	≥ 6.1	≥ 6.1
D						
lipid-lowering treatment						
or TG (mmol/L)		≥ 1.7	≥ 1.7	≥ 1.7	≥ 1.7	≥ 1.7
E						
HDL-C (mmol/L)	male	< 1.03	< 1.03	< 1.03	< 0.9	< 1.04
	female	< 1.29	< 1.29	< 1.29	< 1.0	< 1.04
F						
BMI (kg/m2)		N/A	N/A	N/A	≥ 25	N/A

body mass index (BMI); type 2 diabetes mellitus (T2DM); fasting plasma glucose (FPG); triglycerides (TG); high-density lipoprotein cholesterol (HDL-C); Not applicable (N/A).

IDF criteria: item A is the prime item, plus the presence of at least two items from B to E.

ATP III, JIS, CDS DM criteria: the presence of at least three items from A to E.

CDS criteria: the presence of at least three items from B to F.

### *H. pylori* infection test

*H. pylori* infection was determined using the ^13^C urea breath tests (Beijing Boran Pharmaceutical Co., Ltd., Beijing, China). We followed a standardized procedure for the sample collection. All subjects fasted overnight for more than 8 h, maintained normal breath, inserted the straw into the bottom of one sample tube, and exhaled slowly into the sample tube through the straw for 4 to 5 s. Thereafter, they pulled the straw out, tightened the cap immediately, and this was considered as the 0 min sample. Then, the subjects took another bottle with urea ^13^C granules and 80 to 100 mL cold drinking water, rested for 30 min, and then collected the breath sample again. The two collected gas samples were tested for ^13^CO_2_, and δ‰ was used to represent the determination result. δ‰ = (isotopic abundance of ^13^C for the test sample ─ isotopic abundance of ^13^C for reference sample) × 1000/isotopic abundance ^13^C for reference sample. The detection value was defined as δ‰ measured at 30 min subtracted from that measured at 0 min. *H. pylori* infection was considered positive when the detection value was ≥ 4.0.

### Statistical analysis

IBM SPSS (version 21.0; IBM Corp., NY, USA) was used to perform the statistical analysis. Continuous data are expressed as mean ± standard deviation (SD) for normally distributed data and median with 25^th^ and 75^th^ percentiles for non-normally distributed data. Categorical data are expressed as percentages. The significant differences were evaluated using either the Student’s *t*-test (for continuous variables) or the chi-square test (for categorical variables). Significant and independent predictors of MetS and its components were identified using the multivariable logistic regression analysis. The models were generated wherein the covariates included age, drinking, smoking (Model 1), or Model 1 plus ALT, AST, GGT, creatinine (Model 2), and modeling was performed using the backward stepwise selection methods of variables. The results are expressed as odds ratios (*ORs*) and 95% confidence intervals (*CIs*). Statistical significance was set at *p* < 0.05.

## Results

### Characteristics of study subjects

**Figure 1:**
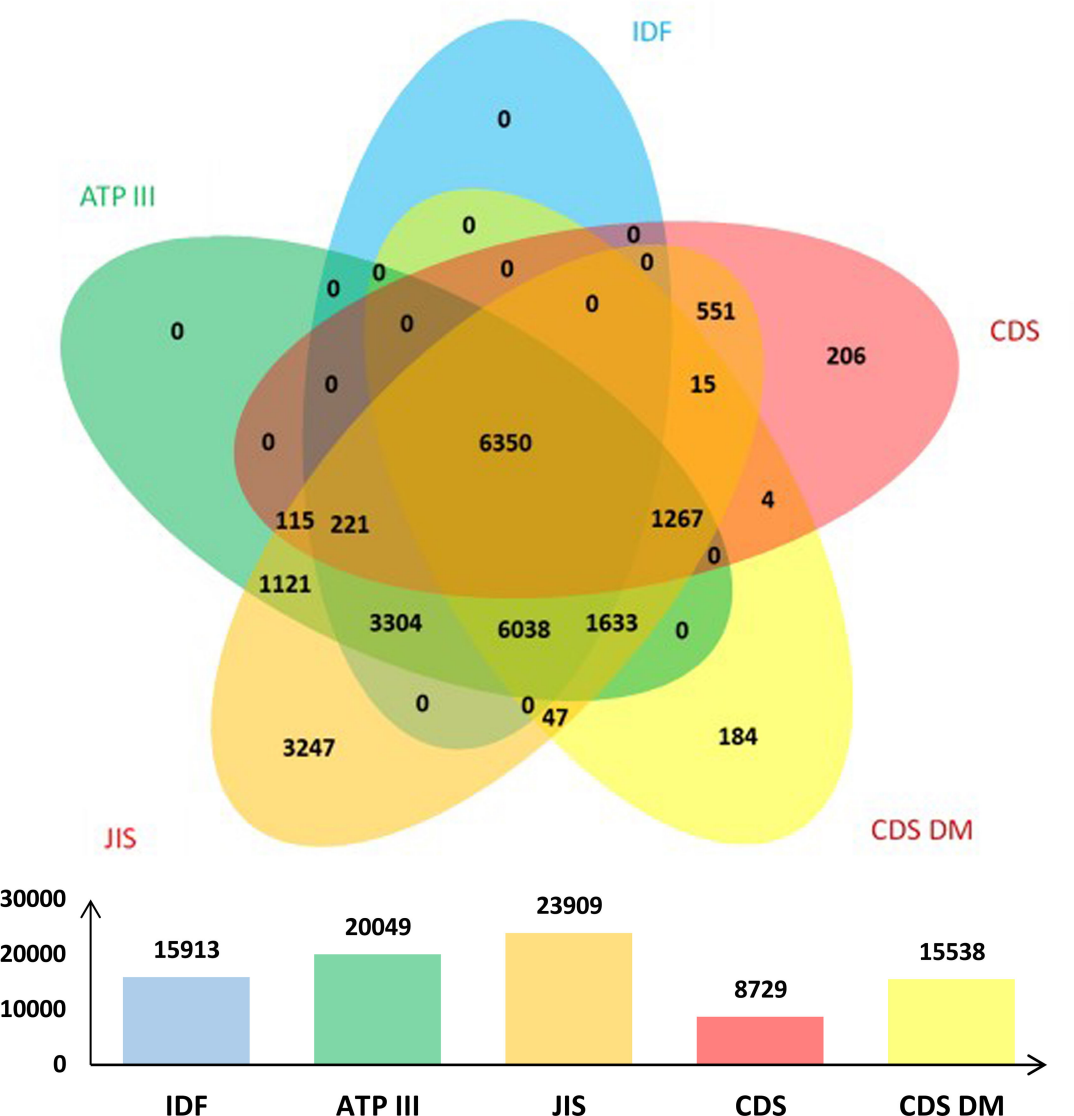
The overlap of MetS defined using the five criteria.

The data of 100,708 subjects (56,301 males and 44,407 females) were obtained in this study, with an average age of 44.76 ± 12.69 years, ranging between 18 to 95 years. The prevalence of MetS according to IDF, ATP III, JIS, CDS and CDS DM criteria was 15.8% (18.7% in males and 12.1% in females), 19.9% (24.3% in males and 14.3% in females), 23.7% (31.2% in males and 14.3% in females), 8.7% (12.0% in males and 4.4% in females), and 15.4% (22.5% in males and 6.4% in females), respectively. The overall infection rate of *H. pylori* was 39.6%, which was higher in males than in females (40.8% *vs*. 38.2%, *p* < 0.001). These results are presented in **Table 2**. The overlap of MetS defined using the five criteria was shown in **Figure 1**.

**Table 2:** Baseline characteristics of the study population (n=100,708).

Variables	All (n=100,708)	Male (n=56,301)	Female (n=44,407)	*p* value
Demographic data				
Sex (female), n (%)	44,407 (44.1)			
Age (years)	44.76 ± 12.69	45.31 ± 12.67	44.06 ± 12.69	<0.001
Hypertension or SBP ≥ 130 mmHg and/or DBP ≥ 85 mmHg^abce^, n (%)	28,899 (28.7)	19,641 (34.9)	9,258 (20.8)	<0.001
Hypertension or SBP ≥ 140 mmHg and/or DBP ≥ 90 mmHg^d^, n (%)	17,317 (17.2)	11,642 (20.7)	5,675 (12.8)	<0.001
T2DM or FPG ≥ 5.6 mmol/L^abc^, n (%)	15,116 (15.0)	10,328 (18.3)	4,788 (10.8)	<0.001
T2DM or FPG ≥ 6.1 mmol/L^de^, n (%)	8,129 (8.1)	5,912 (10.5)	2,217 (5.0)	<0.001
Dyslipidemia^abc^, n (%)	46,233 (45.9)	29,973 (53.2)	16,260 (36.6)	<0.001
Dyslipidemia^d^, n (%)	36,995 (36.7)	27,166 (48.3)	9,829 (22.1)	<0.001
Dyslipidemia^e^, n (%)	40,546 (40.3)	30,309 (53.8)	10,237 (23.1)	<0.001
Waist circumferences ≥ 90 cm in male and ≥ 80 cm in female^ab^, n (%)	30,337 (30.1)	17,996 (32.0)	12,341 (27.8)	<0.001
Waist circumferences ≥ 85 cm in male and ≥ 80 cm in female^c^, n (%)	43,809 (43.5)	31,468 (55.9)	12,341 (27.8)	<0.001
Waist circumferences ≥ 90 cm in male and ≥ 85 cm in female^e^, n (%)	24,246 (24.1)	17,996 (32.0)	6,250 (14.1)	<0.001
BMI ≥ 25 kg/m^2d^, n (%)	32,558 (32.3)	24,240 (43.1)	8,318 (18.7)	<0.001
MetS^a^, n (%)	15,913 (15.8)	10,545 (18.7)	5,368 (12.1)	<0.001
MetS^b^, n (%)	20,049 (19.9)	13,686 (24.3)	6,363 (14.3)	<0.001
MetS^c^, n (%)	23,909 (23.7)	17,546 (31.2)	6,363 (14.3)	<0.001
MetS^d^, n (%)	8,729 (8.7)	6,784 (12.0)	1,945 (4.4)	<0.001
MetS^e^, n (%)	15,538 (15.4)	12,685 (22.5)	2,853 (6.4)	<0.001
Smoking, n (%)	19,545 (19.4)	19,260 (34.2)	285 (0.6)	<0.001
Drinking, n (%)	8,472 (8.4)	8,349 (14.8)	123 (0.3)	<0.001
Anthropometric data				
Body weight (kg)	63.56 ± 11.83	69.95 ± 10.32	55.45 ± 8.06	<0.001
Height (cm)	163.73 ± 8.30	168.72 ± 6.33	157.40 ± 5.80	<0.001
BMI (kg/m^2^)	23.60 ± 3.31	24.55 ± 3.13	22.40 ± 3.14	<0.001
Waist circumferences (cm)	80.93 ± 10.23	85.66 ± 8.66	74.96 ± 8.82	<0.001
Hip circumferences (cm)	94.44 ± 6.26	96.44 ± 5.84	91.91 ± 5.83	<0.001
Waist-Hip ratio	0.86 ± 0.07	0.89 ± 0.06	0.81 ± 0.07	<0.001
Neck circumferences (cm)	34.65 ± 3.63	37.06 ± 2.61	31.59 ± 2.11	<0.001
Laboratory data				
ALT (U/L)	23 (15, 35)	29 (20, 42)	17 (13, 24)	<0.001
AST (U/L)	26.79 ± 10.23	28.69 ± 10.84	24.38 ± 8.83	<0.001
GGT (U/L)	23 (15, 39)	31 (21, 52)	16 (12, 23)	<0.001
FPG (mmol/L)	5.11 ± 1.26	5.21 ± 1.46	4.97 ± 0.93	<0.001
HbA1c (%)	5.54 ± 0.79	5.62 ± 0.89	5.42 ± 0.63	<0.001
Total cholesterol (mmol/L)	4.82 ± 0.93	4.87 ± 0.93	4.75 ± 0.92	<0.001
Triglycerides (mmol/L)	1.35 (0.93, 2.02)	1.61 (1.11, 2.38)	1.09 (0.80, 1.56)	<0.001
LDL-cholesterol (mmol/L)	2.83 ± 0.80	2.92 ± 0.80	2.71 ± 0.79	<0.001
HDL-cholesterol (mmol/L)	1.34 ± 0.33	1.22 ± 0.29	1.49 ± 0.32	<0.001
Uric acid (μmol/L)	347.21 ± 88.84	394.78 ± 78.04	286.89 ± 60.57	<0.001
Platelet count (10^9^/L)	193.89 ± 59.97	188.88 ± 57.03	200.24 ± 62.93	<0.001
*H. pylori* positive, n (%)	39,930 (39.6)	22,946 (40.8)	16,984 (38.2)	<0.001

systolic pressure (SBP); diastolic pressure (DBP); type 2 diabetes mellitus (T2DM); fasting plasma glucose (FPG); body mass index (BMI); alamine aminotransferase (ALT); aspartate aminotransferase (AST); gamma-glutamyltransferase (GGT); hemoglobin A1c (HbA1c).

IDF criteria; b. ATP III criteria; c. JIS criteria; d. CDS criteria; e. CDS DM criteria.

### Prevalence of *H. pylori* positive and MetS stratified by age

The subjects were stratified based on their age. Among males and females, the prevalence of *H. pylori* infection increased with age and reached the peak in the age group of 50-59 years; however, it somehow declined in individuals after 60 years of age. The prevalence of *H. pylori* infection in males was significantly higher than that in females, except in the age group of 40-49 years. Under the five criteria used for defining MetS, the males exhibited a peak prevalence of MetS in the age group of 50-59 years, which also decreased after 60 years of age; however, in females, it continued to increase with age. The prevalence of MetS in males was higher than that in females before 60 years old (IDF, ATP III and JIS criteria) or 70 years old (CDS and CDS DM criteria), and then lowered compared to females; the difference was statistically significant, as shown in **Table 3**.

**Table 3:** The prevalence of *H. pylori* infection and MetS stratified by age.

age	n	*H.pylori* positive [n (%)]	MetS [n (%)]				
			IDF	ATP III	JIS	CDS	CDS DM
Male							
18-29	5,597	1,894 (33.8)^•^	461 (8.2)^•^	575 (10.3)^•^	791 (14.1)^•^	133 (2.4)^•^	550 (9.8)^•^
30-39	14,050	5,453 (38.8)^•^	2,012 (14.3)^•^	2,524 (18.0)^•^	3,449 (24.5)^•^	803 (5.7)^•^	2,389 (17.0)^•^
40-49	17,306	7,205 (41.6)	3,580 (20.7)^•^	4,605 (26.6)^•^	5,854 (33.8)^•^	2,297 (13.3)^•^	4,288 (24.8)^•^
50-59	11,664	5,227 (44.8)^•^	2,818 (24.2)^•^	3,749 (32.1)^•^	4,645 (39.8)^•^	2,160 (18.5)^•^	3,451 (29.6)^•^
60-69	5,126	2,144 (41.8)^•^	1,111 (21.7)^•^	1,470 (28.7)^•^	1,846 (36.0)^•^	931 (18.2)^•^	1,325 (25.8)^•^
≥70	2,558	1,023 (40.0)^•^	563 (22.0)^•^	763 (29.8)^•^	961 (37.6)^•^	460 (18.0)^•^	682 (26.7)^•^
Chi-square value		220.967	901.065	1440.829	1613.450	1776.857	1203.537
*p* value		<0.001	<0.001	<0.001	<0.001	<0.001	<0.001
Female							
18-29	6026	1,895 (31.4)	114 (1.9)	124 (2.1)	124 (2.1)	7 (0.1)	33 (0.5)
30-39	11,220	4,162 (37.1)	446 (4.0)	512 (4.6)	512 (4.6)	78 (0.7)	154 (1.4)
40-49	13,008	5,314 (40.9)	1,170 (9.0)	1,415 (10.9)	1,415 (10.9)	310 (2.4)	505 (3.9)
50-59	8,642	3,533 (40.9)	1,670 (19.3)	2,022 (23.4)	2,022 (23.4)	630 (7.3)	905 (10.5)
60-69	3,892	1,514 (38.9)	1,297 (33.3)	1,503 (38.6)	1,503 (38.6)	552 (14.2)	769 (19.8)
≥70	1,619	566 (35.0)	671 (41.4)	787 (48.6)	787 (48.6)	368 (22.7)	487 (30.1)
Chi-square value		195.163	4792.178	5736.357	5736.357	3118.637	3855.860
*p* value		<0.001	<0.001	<0.001	<0.001	<0.001	<0.001

• compared with the same age group in female, P <0.05.

### The prevalence of MetS or its components under different *H. pylori* infection status

**Figure 2:**
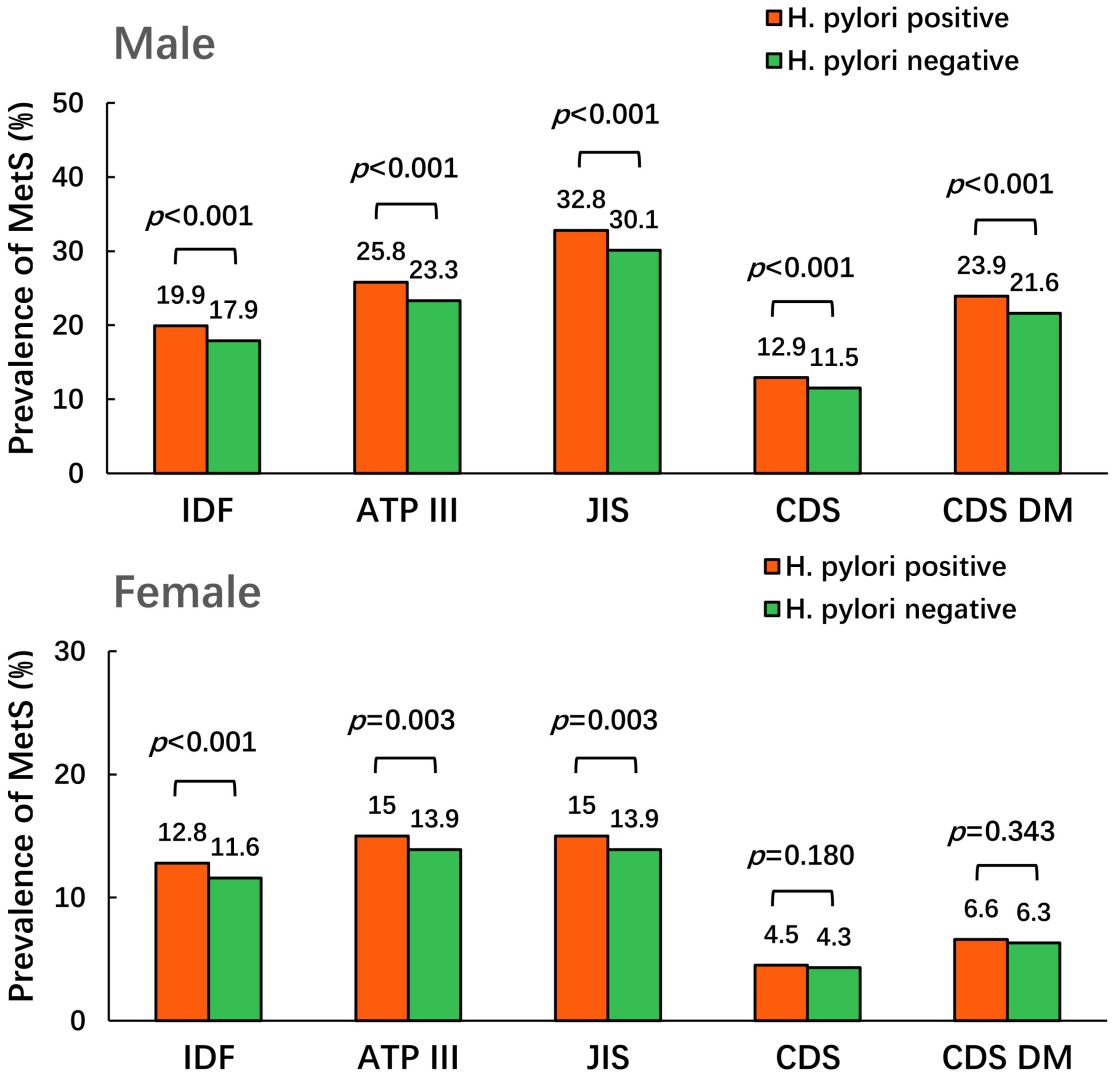
The prevalence of MetS under different *H. pylori* status.

In males, the prevalence of MetS defined using the five criteria in *H. pylori*-positive group was higher than that in the negative group (19.9% *vs*. 17.9% for IDF criteria; 25.8% *vs*. 23.3% for ATP III criteria; 32.8% *vs*. 30.1% for JIS criteria; 12.9% *vs*. 11.5% for CDS criteria; 23.9% *vs*. 21.6% for CDS DM criteria, all *p* < 0.001). In females, the prevalence of MetS defined using the three international criteria in the *H. pylori*-positive group was higher than that in the negative group (12.8% *vs*. 11.6% for IDF criteria, *p* < 0.001; 15.0% *vs*. 13.9% for ATP III and JIS criteria, *p* = 0.003), but no significant differences were observed upon using the two Chinese criteria (4.5% *vs*. 4.3% for CDS criteria, *p* = 0.180; 6.6% *vs*. 6.3% for CDS DM criteria, *p* = 0.343), as shown in **Figure 2**.

**Figure 3:**
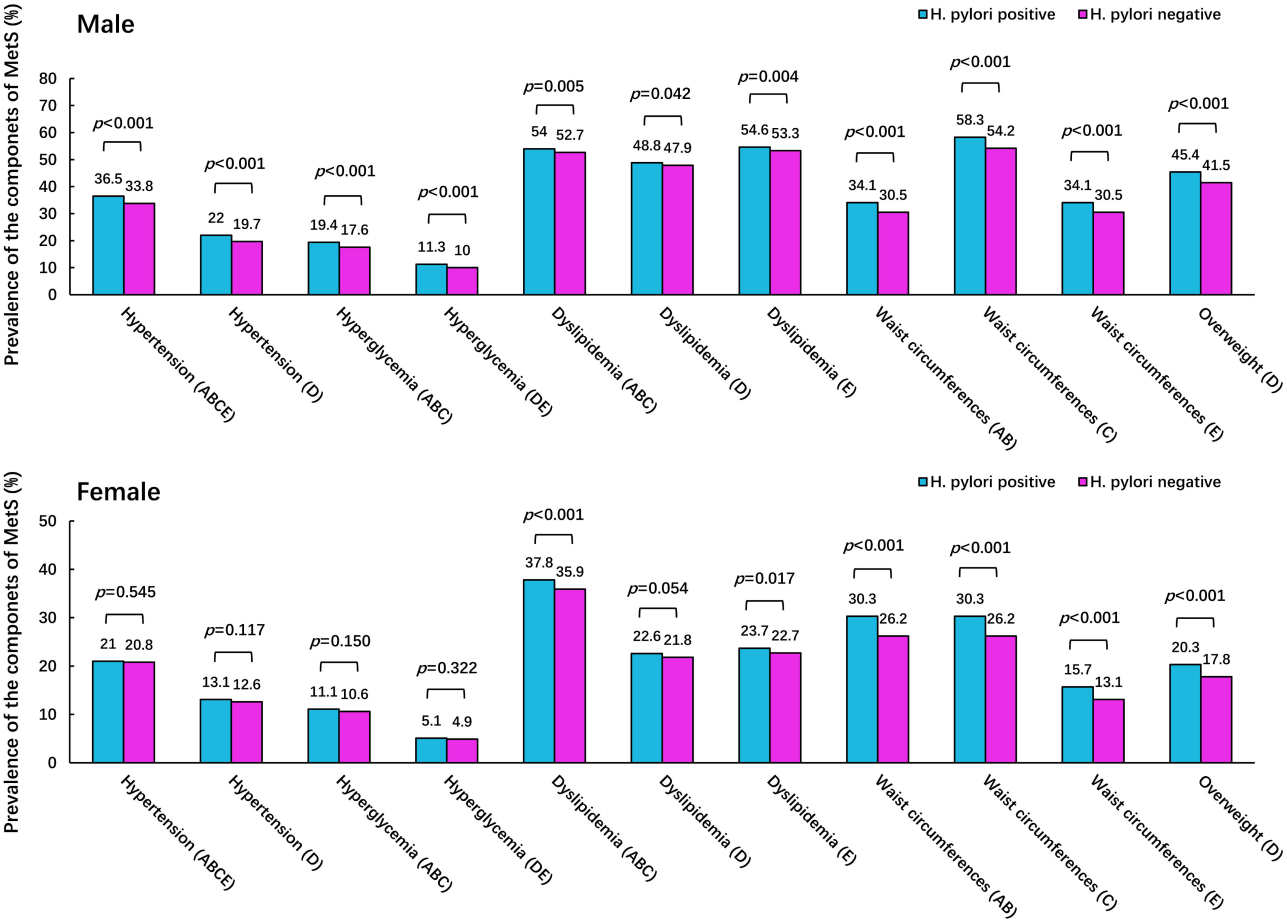
The prevalence of components of MetS under different *H*. *pylori* status. Footnote: The letters in brackets of the abscissa item name represent different criteria of MetS: A. IDF criteria; B. ATP III criteria; C. JIS criteria; D. CDS criteria; E. CDS DM criteria.

The prevalence of MetS components under different *H. pylori* status was also investigated. The results revealed that in males, the prevalence of different components defined using the five criteria of MetS in *H. pylori*-positive group was significantly higher than that in the negative group (all *p* < 0.05). However, in females, the prevalence of excess waist circumference, overweight and dyslipidemia (except the CDS criteria) was higher in the *H. pylori*-positive group than that in the negative group (all *p* < 0.05), but no significant differences were observed in the prevalence of hypertension and hyperglycemia (all *p* > 0.05), as shown in **Figure 3**.

### Logistic regression analysis of *H. pylori* infection with MetS and its components

Univariate logistic regression analysis was performed with MetS defined using the five criteria as the dependent variable. The results revealed that *H. pylori* infection is a potential risk factor associated with MetS defined using the five criteria in males and the three international criteria in females. After adjusting for age, smoking, and drinking, the *OR* for MetS remained significant in males but no longer in females except for the IDF criteria. Furthermore, upon adjusting for ALT, AST, GGT and creatinine, the *OR* for MetS remained significant in males, but *H. pylori* infection was no longer associated with the risk of MetS defined using all the five criteria in females, as shown in **Table 4**.

**Table 4:** Logistic regression analysis of the effect of *H. pylori* infection on MetS defined by different criteria [*OR* (95% *CI*)].

	Not adjusted	Model 1	Model 2
IDF criteria			
male	1.137 (1.089-1.187)	1.100 (1.053-1.149)	1.062 (1.015-1.111)
female	1.114 (1.051-1.180)	1.086 (1.021-1.156)	1.050 (0.985-1.119)
ATP III criteria			
male	1.144 (1.101-1.190)	1.105 (1.062-1.150)	1.069 (1.026-1.114)
female	1.086 (1.028-1.146)	1.053 (0.993-1.117)	1.019 (0.959-1.082)
JIS criteria			
male	1.132(1.092-1.174)	1.095 (1.055-1.136)	1.058 (1.018-1.099)
female	1.086(1.028-1.146)	1.053 (0.993-1.117)	1.019 (0.959-1.082)
CDS criteria			
male	1.139 (1.082-1.199)	1.102 (1.058-1.147)	1.059 (1.004-1.117)
female	1.066 (0.971-1.169)	1.018 (0.937-1.105)	1.025 (0.929-1.132)
CDS DM criteria			
male	1.139 (1.094-1.185)	1.092 (1.036-1.150)	1.066 (1.021-1.112)
female	1.038 (0.961-1.122)	1.057 (0.959-1.165)	0.982 (0.903-1.068)

adjusted for age, smoking, drinking; Model 2: adjusted for age, ALT, AST, GGT, creatinine, smoking, drinking.

The association between *H. pylori* infection and the components of MetS was also investigated using the multivariate logistic regression analysis. After adjusting for age, smoking, drinking, ALT, AST, GGT and creatinine, the results revealed that using the five criteria of MetS, *H. pylori* infection was identified as a risk factor associated with excess waist circumference and BMI in both males and females. *H. pylori* infection was also identified as a risk factor associated with hypertension and hyperglycemia in males, but not in females. No association was identified between *H. pylori* infection and dyslipidemia in both males and females, as shown in **Table 5**.

**Table 5:** Logistic regression analysis of the effect of *H. pylori* infection on the components of MetS defined by different criteria [*OR* (95% *CI*)].

	Not adjusted	Model 1	Model 2
Male			
IDF or ATP III criteria			
Hypertension or SBP ≥ 130 mmHg and/or DBP ≥ 85 mmHg	1.125 (1.086-1.165)	1.073 (1.034-1.114)	1.058 (1.019-1.098)
T2DM or FPG ≥ 5.6 mmol/L	1.121 (1.074-1.171)	1.072 (1.025-1.121)	1.056 (1.009-1.106)
Dyslipidemia	1.050 (1.015-1.086)	1.041 (1.007-1.078)	0.997 (0.962-1.034)
Waist circumferences ≥ 90 cm in male and ≥ 80 cm in female	1.181 (1.139-1.224)	1.149 (1.108-1.191)	1.116 (1.074-1.159)
JIS criteria			
Hypertension or SBP ≥ 130 mmHg and/or DBP ≥ 85 mmHg	1.125 (1.086-1.165)	1.073 (1.034-1.114)	1.058 (1.019-1.098)
T2DM or FPG ≥ 5.6 mmol/L	1.121 (1.074-1.171)	1.072 (1.025-1.121)	1.056 (1.009-1.106)
Dyslipidemia	1.050 (1.015-1.086)	1.041 (1.007-1.078)	0.997 (0.962-1.034)
Waist circumferences ≥ 85 cm in male and ≥ 80 cm in female	1.183 (1.144-1.224)	1.149 (1.110-1.189)	1.112 (1.073-1.154)
CDS criteria			
Hypertension or SBP ≥ 140 mmHg and/or DBP ≥ 90 mmHg	1.150 (1.104-1.199)	1.097 (1.050-1.147)	1.080 (1.033-1.129)
T2DM or FPG ≥ 6.1 mmol/L	1.148 (1.087-1.212)	1.097 (1.037-1.160)	1.082 (1.021-1.146)
Dyslipidemia	1.035 (1.001-1.071)	1.026 (0.991-1.061)	0.979 (0.945-1.015)
BMI ≥ 25 kg/m2	1.173 (1.134-1.214)	1.158 (1.119-1.198)	1.122 (1.082-1.163)
CDS DM criteria			
Hypertension or SBP ≥ 130 mmHg and/or DBP ≥ 85 mmHg	1.125 (1.086-1.165)	1.073 (1.034-1.114)	1.058 (1.019-1.098)
T2DM or FPG ≥ 6.1 mmol/L	1.148 (1.087-1.212)	1.097 (1.037-1.160)	1.082 (1.021-1.146)
Dyslipidemia	1.051 (1.017-1.087)	1.043 (1.008-1.079)	0.999 (0.964-1.035)
Waist circumferences ≥ 90 cm in male and ≥ 85 cm in female	1.181 (1.139-1.224)	1.149 (1.108-1.191)	1.116 (1.074-1.159)
Female			
IDF or ATP III criteria			
Hypertension or SBP ≥ 130 mmHg and/or DBP ≥ 85 mmHg	1.015 (0.968-1.064)	0.957 (0.907-1.010)	0.949 (0.899-1.001)
T2DM or FPG ≥ 5.6 mmol/L	1.046 (0.984-1.113)	1.017 (0.954-1.085)	0.996 (0.932-1.063)
Dyslipidemia	1.087 (1.045-1.131)	1.059 (1.017-1.103)	1.038 (0.996-1.081)
Waist circumferences ≥ 90 cm in male and ≥ 80 cm in female	1.221 (1.170-1.274)	1.187 (1.135-1.242)	1.158 (1.106-1.213)
JIS criteria			
Hypertension or SBP ≥ 130 mmHg and/or DBP ≥ 85 mmHg	1.015 (0.968-1.064)	0.957 (0.907-1.010)	0.949 (0.899-1.001)
T2DM or FPG ≥ 5.6 mmol/L	1.046 (0.984-1.113)	1.017 (0.954-1.085)	0.996 (0.932-1.063)
Dyslipidemia	1.087 (1.045-1.131)	1.059 (1.017-1.103)	1.038 (0.996-1.081)
Waist circumferences ≥ 85 cm in male and ≥ 80 cm in female	1.221 (1.170-1.274)	1.187 (1.135-1.242)	1.158 (1.106-1.213)
CDS criteria			
Hypertension or SBP ≥ 140 mmHg and/or DBP ≥ 90 mmHg	1.047 (0.989-1.108)	1.024 (0.959-1.093)	1.012 (0.947-1.081)
T2DM or FPG ≥ 6.1 mmol/L	1.045 (0.958-1.141)	1.032 (0.941-1.130)	1.006 (0.917-1.104)
Dyslipidemia	1.046 (0.999-1.095)	1.008 (0.962-1.057)	0.986 (0.939-1.035)
BMI ≥ 25 kg/m2	1.181 (1.125-1.240)	1.150 (1.094-1.209)	1.112 (1.056-1.171)
CDS DM criteria			
Hypertension or SBP ≥ 130 mmHg and/or DBP ≥ 85 mmHg	1.015 (0.968-1.064)	0.957 (0.907-1.010)	0.949 (0.899-1.001)
T2DM or FPG ≥ 6.1 mmol/L	1.045 (0.958-1.141)	1.032 (0.941-1.130)	1.006 (0.917-1.104)
Dyslipidemia	1.057 (1.010-1.106)	1.019 (0.973-1.068)	0.997 (0.950-1.046)
Waist circumferences ≥ 90 cm in male and ≥ 85 cm in female	1.241 (1.176-1.311)	1.217 (1.150-1.288)	1.180 (1.113-1.251)

Model 1: adjusted for age, smoking, drinking; Model 2: adjusted for age, ALT, AST, GGT, creatinine, smoking, drinking.

## Discussion

*H. pylori* infection and MetS are two important public health issues in the contemporary society. Some researchers attempted to discussed the correlation between these two, but the results are inconsistent and conflicting. Gunji et al. investigated the data of 7,394 Japanese subjects and suggested that there was a significant and independent correlation between the positive rate of serum *H. pylori* antibody and MetS (12). Lim et al. conducted an international multicenter study including 15,195 Korean subjects, and suggested that *H. pylori* infection plays an independent role in the development of MetS in Korean people under 65 years of age (15). However, inconsistent results have also been reported. Takeoka et al. investigated 1,044 Japanese subjects, and found no significant association between *H. pylori*-seropositive status and the risk of MetS upon adjusting for age and sex (14). Naja et al. reported no association between *H. pylori* seropositivity and MetS or IR among 308 Lebanese adults (13). The inconsistency in the results was thought to be partly due to the different criteria used for defining MetS (16).

In our study, five MetS criteria were used for the analysis to avoid the impact of using different criteria on the results. We found that in males, the prevalence of MetS defined using the five criteria was higher in the *H. pylori*-positive group than in the negative group. In females, the prevalence of MetS defined using the three international criteria in *H. pylori*-positive group was higher than that in the negative group, but the differences were not significant using the two Chinese criteria. According to the multivariate regression analysis upon adjusting for age, smoking, drinking, ALT, AST, GGT and creatinine, we found that *H. pylori* positivity was still a risk factor associated with MetS defined using the five criteria in males.

However, the results based on gender differences in our study were different from those of previous similar studies, which suggest that *H. pylori* infection increased the risk of MetS in females (22, 23). We further analyzed the relationship between *H. pylori* infection and different MetS components. The results of the multivariate regression analysis revealed that *H. pylori* positivity was a risk factor associated with excess waist circumference and BMI in both males and females upon using the five criteria for defining MetS. Furthermore, *H. pylori* positivity was also identified as a risk factor associated with hypertension and hyperglycemia in males, but not in females. No correlation was identified between *H. pylori* positivity and dyslipidemia in either males or females.

As well known, IR forms the pathophysiological basis of MetS, and growing evidences have shown that *H. pylori* infection is associated with IR (24, 25). Some researchers thought that *H. pylori* infection caused IR through insulin dysfunction in the liver, which is an important target organ for insulin, *via* the c-Jun/microRNA203/suppressor of cytokine signaling 3 pathway (11). Additionally, the immune response to *H. pylori* infection has also been shown to increase the levels of some proinflammatory cytokines, such as IL-1, IL-6, and TNFa, which causes the phosphorylation of serine/threonine residues on the insulin receptor, disrupt the activation of the receptor, and eventually reduce insulin sensitivity (10, 26). Furthermore, leptin, ghrelin, fetuin A and monocyte chemoattractant protein-1 (MCP-1) are related to the IR process. During *H. pylori* infection, the intestinal hormones and protein molecules mentioned above exhibited the corresponding changes in their levels that contributed in the process of IR promotion (27–30). *H. pylori* infection also induced the production of reactive oxygen species (ROS) and platelet activation, which plays a critical role in IR development (16). Therefore, we hypothesized that *H. pylori* infection may participate in the development of MetS through IR promotion as the underlying mechanism. The improvement of waist circumference, fasting blood glucose, glycosylated hemoglobin, and high-density lipoprotein in MetS patients after radical treatment of *H. pylori* infection also supported its participation in the process of MetS development (31).

IR is also a mechanism underlying the development of hyperglycemia. However, it is worth noting that in our study, we found a correlation between hyperglycemia and *H. pylori* infection only in males. Previous studies have demonstrated gender differences in IR and suggested that females exhibited less insulin resistance (32, 33). Androgen itself appeared to contribute to IR development (34). This difference in IR between the males and females would be further reflected through the effect on serum glucose levels.

Similarly, there was a clear gender difference in the correlation between *H. pylori* infection and hypertension. Moreover, the prevalence of hypertension was different between males and females, and males more likely developed hypertension (35). As for the mechanisms that would link *H. pylori* infection to hypertension, it was thought to be related to the increase in the levels of many inflammatory factors induced upon *H. pylori* infection. These inflammatory factors are considered to play important roles in the pathogenesis of atherosclerosis and reduce the elasticity of blood vessels (36). IR itself has also been reported to increase the peripheral blood pressure (37). Animal experiments demonstrated the association of hyperinsulinemia and insulin resistance with hypertension in male only (38). Therefore, the correlation between IR and hypertension appears to be sex-dependent, and more attention should be paid to the improvement of hypertension and hyperglycemia in males upon *H. pylori* infection.

In our study, the results of waist circumference and BMI suggested a correlation between obesity and *H. pylori* infection in both males and females. This might be different from hypertension and hyperglycemia, which mainly occur as a result of IR, and the relationship between IR and obesity was more complex, for being a mutual cause and effect scenario (39). IR occurred as long as obesity was present, while hypertension and hyperglycemia required IR to reach a certain level. This difference in the cause-and-effect relationship might also be observed in case of various metabolic disorders caused by IR due to *H. pylori* infection.

Dyslipidemia is closely related to IR. Previous studies have suggested that *H. pylori* infection may cause dyslipidemia (40), and indicated that *H. pylori* infection was significantly and independently associated with dyslipidemia (41). However, conflicting results are also present (42). In our study, we did not find a correlation between *H. pylori* infection and dyslipidemia in both males and females. Whether there is a correlation between *H. pylori* infection and dyslipidemia remains to be elucidated yet.

The limitation of our study was that the subjects were from the health examination population rather than from random sampling of the community, which led to sample deviation, but this was somewhat compensated through the inclusion of relatively large number of subjects. Second, our study lacked socioeconomic data, which might have led to the exclusion of confounding factors. However, the prevalence of *H. pylori* was similar to that reported in a previous study (22), and the bias might not be significant. Moreover, this cross-sectional study might only reflect the association between *H. pylori* infection and MetS, rather than causality. The pathogenic mechanisms of *H. pylori* infection and MetS are diverse and complex. Moreover, MetS is considered as a chronic inflammatory or proinflammatory state, and cytokines induced upon *H. pylori* infection have also been observed in MetS (43). Even in the process of MetS caused due to *H. pylori* infection, MetS itself or its components could be correlated to the status of *H. pylori* infection through multiple mechanisms. This may lead to an interaction or even a vicious circle between them, thereby increasing the complexity of the result determination. Only through the study of specific biological mechanism, we may effectively elucidate the relationship between *H. pylori* infection and MetS and its components.

## Conclusions

Conclusively, our study suggests that *H. pylori* infection is associated with MetS in males. Additionally, *H. pylori* infection was found to be correlated with MetS components (such as hyperglycemia, hypertension, obesity, etc.) in males. As a warning, it might be needed for males with *H. pylori* infection to pay greater attention to weight control and proper diet. However, these results need to be verified further through more clinical studies, especially cohort ones with high sample sizes. Authoritative and credible research conclusions may provide a new strategy for treating MetS in males.

## Data availability statement

The raw data supporting the conclusions of this article will be made available by the authors, without undue reservation.

## Ethics statement

The studies involving human participants were reviewed and approved by Ethics committees of the Sichuan Provincial People’s Hospital. Written informed consent for participation was not required for this study in accordance with the national legislation and the institutional requirements.

## Author contributions

YiL and YuL designed the study. YiL, WC and DL wrote the manuscript. YiL and PS performed the data collection and analysis. YuL assisted in data collection and document writing. All authors read and approved the manuscript.
